# *GnRH *and *LHR *gene variants predict adverse outcome in premenopausal breast cancer patients

**DOI:** 10.1186/bcr1756

**Published:** 2007-08-10

**Authors:** Djura Piersma, Axel PN Themmen, Maxime P Look, Jan GM Klijn, John A Foekens, André G Uitterlinden, Huibert AP Pols, Els MJJ Berns

**Affiliations:** 1 Department of Internal Medicine, Erasmus MC, 3000 CA Rotterdam, The Netherlands; 2 Department of Medical Oncology, Erasmus MC, 3000 CA Rotterdam, The Netherlands; 3 Department of Epidemiology and Biostatistics, Erasmus MC, 3000 CA Rotterdam, The Netherlands

## Abstract

**Background:**

Breast cancer development and progression are dependent on estrogen activity. In premenopausal women, estrogen production is mainly regulated through the hypothalamic-pituitary-gonadal (HPG) axis.

**Methods:**

We have investigated the prognostic significance of two variants of genes involved in the HPG-axis, the *GnRH *(encoding gonadotropin-releasing hormone) *16Trp/Ser *genotype and the *LHR *(encoding the luteinizing hormone receptor) *insLQ *variant, in retrospectively collected premenopausal breast cancer patients with a long follow-up (median follow-up of 11 years for living patients).

**Results:**

Carriership was not related with breast cancer risk (the case control study encompassed 278 premenopausal cases and 1,758 premenopausal controls). A significant adverse relationship of the *LHR insLQ *and *GnRH 16Ser *genotype with disease free survival (DFS) was observed in premenopausal (hormone receptor positive) breast cancer patients. In particular, those patients carrying both the *GnRH 16Ser *and *LHR insLQ *allele (approximately 25%) showed a significant increased risk of relapse, which was independent of traditional prognostic factors (hazard ratio 2.14; 95% confidence interval 1.32 to 3.45; *P *= 0.002).

**Conclusion:**

We conclude that the *LHR insLQ *and *GnRH 16Ser *alleles are independently associated with shorter DFS in premenopausal patients. When validated, these findings may provide a lead in the development of tailored treatment for breast cancer patients carrying both polymorphisms.

## Introduction

The diagnosis of breast cancer is made one million times each year worldwide. About one-quarter of these women are premenopausal at time of diagnosis, which is associated with poor prognosis compared to postmenopausal women [1,2]. It is anticipated that, as a result of changing demographic and lifestyle factors, more and more women will be diagnosed at a younger age with breast cancer [3,4]. In addition to age and family history, several factors relating to increased or prolonged cumulative estrogen exposure have been identified as important risk factors for breast cancer development and progression [5,6]. Polymorphic variation in genes regulating estrogen production may partly explain differences in susceptibility, clinical presentation and outcome of breast cancer between individuals or populations [6,7].

In premenopausal women, estrogens predominantly arise from the ovaries, where production is regulated by the neuro-endocrine system consisting of hypothalamus, pituitary and gonads: the HPG axis. Internal and external stimuli are integrated in the brain, resulting in the pulsatile secretion of the hypothalamic neuropeptide gonadotropin-releasing hormone (GnRH). GnRH reaches the gonadotroph cells in the anterior pituitary through the hypophysial portal circulation, where it stimulates *de novo *synthesis and secretion of the gonadotropins follicle stimulating hormone and luteinizing hormone (LH), which reach the ovaries in women through the circulation. LH, acting through the LH receptor (LHR), stimulates production of androgen precursors in theca cells that surround antral follicles. Follicle stimulating hormone subsequently regulates the granulosa cell enzyme aromatase, which converts these androgens to estrogens. In turn, ovarian sex steroid and peptide hormones (inhibin A and B) provide negative feedback regulation, either in the pituitary or hypothalamus. The menopausal transition, that is, the cessation of menstrual cycling, is characterized by disruption of this tightly balanced HPG axis system and is accompanied by continuously increased serum LH and follicle stimulating hormone in combination with decreased levels of ovarian sex steroid hormones [8,9].

We have previously reported, in a training set of 266 Australian breast cancer patients, an association between a common polymorphic CTCCAG (Leu-Gln (LQ)) insertion (*LHR insLQ*) in the signal peptide of the *LHR *gene and poor survival [10]. No associations between its ligand, LH, genotype and clinical parameters were observed in this study. In our subsequent validation study on a large independent breast cancer cohort of 751 retrospectively collected Dutch patients with long detailed follow-up, we have confirmed the association of the *LHR insLQ *gene variant with a shorter disease-free survival (DFS) [11]. Furthermore, we have shown the functional importance of the LQ insertion in the signal peptide, that is, an increased activity for the LHR insLQ variant compared with the LHR non-LQ protein. We hypothesized an ovary-dependent increase in cumulative estrogen exposure that may influence breast cancer outcome in patients with the *LHR insLQ *genotype [10]. Interestingly, the *GnRH *gene also carries a common signal peptide polymorphism (Trp16Ser) [12], which has been associated with altered bone mineral density, an indirect marker for estrogen exposure [13].

In line with the hypothesis that possible associations of the above mentioned polymorphisms with outcome would depend on HPG-regulation of ovarian function, we have investigated associations of the *LHR insLQ *and *GnRH 16Ser *alleles with premenopausal breast cancer outcome in the present study.

We observed that hormone receptor positive premenopausal women carrying either of the variant alleles or the combined variant alleles of both genes had a significant shorter DFS; *LHR insLQ *and the combined alleles were independent of traditional prognostic factors.

## Materials and methods

### Breast tumor samples

The study design was approved by the Medical Ethics Committee of the Erasmus MC, Rotterdam, the Netherlands. From the DNA samples with complete follow up described previously [11], we have included 278 premenopausal patients with known estrogen receptor (ER) status, conclusive genotypes and complete follow up. All tumors were invasive (42 had an additional *in situ *component).

The menopausal status of patients was determined according to the guidelines of the European Organization of Research and Treatment of Cancer (EORTC). The median age of patients at diagnosis was 45 years (range 22 to 57 years). The median follow-up period of all patients was 112 months (range 9 to 255 months) and of still living patients 130 months from primary surgery (range 13 to 255 months). Pathological examination was not performed centrally and reflects daily practice in the various participating regional hospitals as described previously. Other patient characteristics are listed in Table 1.

**Table 1:** Baseline characteristics in 278 premenopausal patients according to *LHR insLQ *and *GnRH 16Ser *carriership

		*LHR insLQ*			*GnRH 16Ser*		
					
Feature	Number (percent)	Non-carriers (percent)	Carriers (percent)^a^	*P *value^a^	Non-carriers (percent)	Carriers (percent)	*P *value^a^
**Breast cancer cases**	278 (100)	147 (53)	131 (47)		157 (56)	121 (44)	
**Age in years**				0.18			0.56
<35	19 (7)	12	7		9	10	
35–39	35 (12)	13	22		17	18	
40–49	167 (60)	93	74		99	68	
50–59	57 (21)	29	28		32	25	
**Node status**				0.78			**0.001^b^**
Negative	134 (48)	72	62		89	45	
Positive	144 (52)	75	69		68	76	
**Histological grade^c^**				**0.04**			0.15
Well/mod	66 (24)	44	22		41	25	
unknown	59 (21)	28	31		27	32	
Poor	153 (55)	75	78		89	64	
**Tumor size^d^**				0.58			0.30
≤2 cm	113 (41)	62	51		68	45	
>2 cm	165 (59)	85	80		89	76	
**Estrogen receptor status^c,e^**				0.095			0.29
Negative	78 (28)	35	43		48	30	
Positive	200 (72)	112	88		109	91	
**Progesterone receptor status^c,e^**				0.15			0.54^f^
Negative	70 (25)	32	38		42	28	
Positive	199 (72)	111	88		111	88	
**HER2 amplified^c^**				0.68			0.85
Yes	55 (20)	28	27		30	25	
No	202 (73)	106	96		114	88	
**Adjuvant therapy**				0.54			0.09
No	158 (57)	88	70		96	62	
Hormonal	4 (1)	1	3		1	3	
Chemotherapy	110 (40)	55	55		55	55	
Combined	6 (2)	3	3		5	1	

^a^χ^2 ^test. ^b^Also, when stratified per 'genotype', *P *= 0.003. ^c^Numbers in cells may not add up due to incomplete information on histological grade and/or receptor status. ^d^Tumors of unknown size (*n *= 10) are included as tumor size >2 cm. ^e^The cutoff value used was 10 fmol/mg protein. ^f^Significant when stratified per 'genotype', *P *= 0.02. Entries in bold represent significant outcomes.

### Control population

As a control cohort we studied banked blood samples of pre- and perimenopausal women from the Eindhoven Perimenopausal Osteoporosis Study (EPOS). The EPOS study is a population-based cohort study of pre-, peri- and postmenopausal women born between 1941 and 1947 living in the city of Eindhoven, The Netherlands (median age 50.0 years (range 46 to 57 years)). The study rationale and design have been described previously [14]. Participants gave their written informed consent, and the study was approved by the Medical Ethics Committee of the Erasmus MC, Rotterdam. For the present study we included 1,758 (successful genotyping for the *LHR *as well as *GnRH *genotypes) pre- and perimenopausal subjects, after excluding women with a history of breast carcinoma. All subjects (median age 49.5 years) are of Caucasian Dutch descent. Data for baseline examination and blood samples for extracting DNA from peripheral leucocytes were collected between 1994 and 1995.

### Genotyping

High molecular weight genomic DNA was used as a template for PCR amplification. Exon 1 of the *LHR *gene was amplified as described by Atger and colleagues [15] using a 5'-hexachlorofluorescein labeled forward primer. Separation and sizing of the PCR fragments and assignment of *LHR insLQ *genotype was performed using the ABI Prism 3100 automated capillary DNA sequencer and Genescan and Genotyper software packages (Applied Biosystems, Perkin Elmer, Nieuwerkerk aan den IJssel, The Netherlands) as described by us before [11].

The *16Trp/Ser *polymorphism in the *GnRH *gene was determined using the Taqman allelic discrimination assay. Primer sequences used for amplification of the fragment of exon 1, including the single nucleotide polymorphism (SNP) were AATTCAAAAACTCCTAGCTGGCCTTA (forward) and CATAGGACCAGTGCTGGCT (reverse). Used probes (with SNP underlined) were 5'-VIC-CACGCACCAAGTCA (anti-sense) and 5'-FAM-AGCCACGAAGTCA (anti-sense). Primer and probe sequences were optimized using the SNP assay-by-design service of Applied Biosystems (for details, see [16]). These reactions were performed on the Taqman Prism 7900 HT 384 wells format. Snap frozen primary breast cancer specimens, stored in liquid nitrogen and from which the DNA was obtained for genotyping, contain a relatively high proportion (>40%) of non-tumor tissue. This ensures accurate genotyping, irrespective of the possible loss of heterozygosity that may occur in tumor tissue [10]. Furthermore, to test for possible loss of heterozygosity we have examined the Hardy-Weinberg equilibrium (HWE).

### Statistical analysis

Pearson's χ^2 ^analysis and Fisher's exact test were used to test for independence of the alleles (HWE) and for association analyses with patient and tumor characteristics, respectively. We allowed for three possible genetic models to explain differences in patient and tumor characteristics between genotype groups: linear, dominant or recessive effects. A linear effect, assuming a dose-response relationship of the association for the presence of zero, one or two copies of the allele, was tested using a (χ^2^) linear trend analysis [17]. A dominant effect between hetero- and homozygous combined carriers versus non-carriers was tested using χ^2 ^analysis. Indications for recessive effects were not observed. Univariate and multivariate DFS analyses (endpoint: recurrence excluding second primary breast tumor) were carried out using Cox proportional hazards regression analysis. Hazard ratios (HRs) for the *LHR insLQ *and *GnRH 16Ser *alleles are presented with their 95% confidence interval (CI). Differences between HRs per *LHR insLQ *and *GnRH 16Ser *genotype were tested using the likelihood ratio test associated with the Cox regression analysis. In multivariate analysis, Cox proportional hazard models for DFS were applied to test the genotype variables against traditional factors using a forward stepwise model. The multivariate model included age, positive versus negative nodal status, differentiation grade, tumor size (larger tumors versus tumors ≤2 cm), ER status and adjuvant therapy. DFS curves were generated using the actuarial method of Kaplan-Meier [18] and log-rank tests were used to test for equality of survival functions. All computations were carried out using the STATA statistical package, version 9.2 (Stata Corp., College Station, TX, USA). Statistical significance was assumed at *P *≤ 0.05; *P *values are two-tailed and relate to data during the total period of follow-up.

## Results

### *LHR insLQ *and *GnRH 16Trp/Ser *genotyping

Genotype analysis for the *LHR insLQ *polymorphism in the 278 premenopausal patients studied revealed an allele frequency of 0.27. This resulted in 147 (52.9%) *nonLQ/nonLQ *homozygotes, 113 (40.7%) heterozygotes and 18 (6.5%) *insLQ/insLQ *homozygotes. The allele frequency for the *GnRH 16Ser *polymorphism was 0.25. The genotype distribution was 157 (56.5%) *16Trp/16Trp *homozygotes, 103 (37.1%) heterozygotes and 18 (6.5%) *16Ser/16Ser *homozygotes. Both genotypes were found to be in HWE (*P *= 0.55 and *P *= 0.84, respectively). The genotype distributions and allele frequencies in the control cohort from the EPOS study (*n *= 1,758), did not differ significantly from the case distributions. Genotype results for the *LHR insLQ *polymorphism revealed an allele frequency of 0.29. The genotype distribution was 901 (51.3%) *nonLQ/nonLQ *homozygotes, 708 (40.3%) heterozygotes and 149 (8.5%) *insLQ/insLQ *homozygotes. Genotyping for the *GnRH 16Ser *allele resulted in an allele frequency of 0.25 and revealed 1,004 (57.1%) *16Trp/16Trp *homozygotes, 627 (35.7%) heterozygotes and 127 (7.2%) *16Ser/16Ser *homozygotes, in HWE. All genotype frequencies and allele frequencies are closely similar in the cases series and in the population controls, which lends support to the genotyping results in tumor material. We conclude that neither *LHR insLQ *nor *GnRH 16Ser *genotypes influence the risk of breast cancer development.

### Associations with patient and tumor characteristics

The distribution of clinico-pathological characteristics across the *GnRH 16Trp/Ser *genotype showed a dominant effect of presence of the *GnRH 16Ser *allele. Carriers of the *GnRH 16Ser *allele were, therefore, compared to non-carriers. Carriership of the *GnRH 16Ser *allele was significantly associated with increased lymph node involvement (*P *= 0.001), while *GnRH *genotype was associated with progesterone receptor levels (*P *= 0.02). *LHR insLQ *was associated with histological grade. Both polymorphisms were not significantly associated with other clinico-pathological characteristics. No significant interaction between the presence of the *LHR insLQ *and *GnRH 16Ser *variants was observed in these association analyses.

### Associations of *LHR insLQ *and *GnRH 16Ser *variants with DFS

We hypothesized that HPG-mediated increases in cumulative ovarian estrogen exposure influence breast cancer outcome. The adverse association of the *LHR insLQ *allele with DFS was observed in the premenopausal patients (HR for carriers versus non-carriers = 1.59, 95% CI 1.14 to 2.23, *P *= 0.007; Table 2). In these premenopausal patients the *LHR insLQ *genotype was an independent prognostic factor: addition of *LHR insLQ *carriership to the multivariate model resulted in an increase of χ^2 ^from 44.06 to 52.23 (Δχ^2 ^= 8.17 (df = 1), *P *= 0.004) for DFS. The association between the presence of the *GnRH 16Ser *allele and DFS was also tested. An increased HR of 1.40 (95% CI 1.00 to 1.96, *P *= 0.05; Table 2) for *GnRH 16Ser *carriers versus non-carriers was observed.

**Table 2:** Cox univariate and multivariate analysis for disease free survival in the 278 premenopausal breast cancer patients

	Patients		Univariate analysis			Multivariate analysis		
			
Factor of base model	Number	Percent	HR	95 percent CI	P value	HR	95 percent CI	P value
**Age (years)**								
<39	54	24	1.00			1.00		
40–49	167	60	0.72	0.50–1.06		0.66	0.44–0.98	
50–59	57	21	0.42	0.23–0.77	0.013	0.35	0.19–0.67	<0.001
**Nodal status**								
Negative	134	48	1.00			1.00		
Positive	144	52	1.90	1.34–2.70	<0.001	3.68	2.11–6.42	<0.001
**Histological grade**								
Poor	153	55	1.00			1.00		
Unknown	59	21	0.51	0.32–0.83		0.54	0.33–0.88	
Well/moderate	66	24	0.55	0.36–0.85	0.002	0.56	0.35–0.88	0.019
**Tumor size**								
≤2 cm	113	41	1.00			1.00		
>2 cm	165	59	1.77	1.23–2.54	0.002	1.57	1.07–2.30	0.021
**Estrogen receptor status**								
Negative	78	28	1.00			1.00		
Positive	200	72	0.87	0.60–1.27	0.48	1.01	0.68–1.49	0.96
**Adjuvant therapy**								
No	158	57	1.00			1.00		
Yes^a^	120	43	1.41	1.01–1.97	0.045	0.41	0.24–0.70	0.001
**Factors analyzed**								
**Carriership**								
Non-carriersb			1.00			1.00		
GnRH 16Ser	121	44	1.40	1.00–1.96	0.050	1.32	0.93–1.88	0.12
LHR insLQ	131	47	1.59	1.14–2.23	0.007	1.64	1.16–2.32	0.005
**Combined carriership**								
Non-carriers	87	31	1.00			1.00		
Only *GnRH 16Ser*	60	22	1.39	0.84–2.29	0.19	1.24	0.74–2.08	0.42
Only LHR insLQ	70	25	1.59	0.99–2.54	0.055	1.56	0.97–2.51	0.067
*LHR insLQ*+*GnRH16Ser*	61	22	2.17	1.36–3.48	0.001	2.14	1.32–3.45	0.002

^a^Of 120 patients who received adjuvant therapy, 110 received chemotherapy (mainly CMF, *n *= 101), 4 endocrine therapy, and 6 both; node negatives were not treated. ^b^The number of *GnRH 16Ser *non-carriers was 157, and of *LHR insLQ *non-carriers was 147 patients.

Interestingly, in the biological relevant hormone receptor subgroup (ER and/or progesterone receptor positive, *n *= 225 (81%)), the *LHR insLQ *genotype retained significance. The adverse association of the *LHR insLQ *allele with DFS had a HR for carriers versus non-carriers of 1.59 (95% CI 1.11 to 2.28, *P *= 0.012). It was also an independent prognostic factor: addition of *LHR insLQ *carriership to the multivariate model resulted in an increase of χ^2 ^from 36.28 to 42.70 (Δχ^2 ^= 6.42 (df = 1), *P *= 0.01) for DFS. Moreover, the association between the presence of the *GnRH 16Ser *allele and DFS revealed a significantly increased HR of 1.44 (95% CI 1.01 to 2.07, *P *= 0.046). In multivariate analysis for *GnRH 16Ser *carriers versus non-carriers this was not significant.

### Cooperative effect of variants on the HPG axis and DFS

The HPG system in the premenopausal woman involves a cooperative effect of both GnRH and LH action on the regulation of ovarian sex steroid production. Therefore, we have examined in an exploratory study the combined effect of the *GnRH 16Ser *and *LHR insLQ *variants in these premenopausal breast cancer patients. We combined heterozygous and homozygous carriers, providing four groups of similar sizes. Non-carriers, carriers of the *GnRH 16Ser *allele, carriers of the *LHR insLQ *allele, and carriers of both alleles were compared.

**Figure 1:**
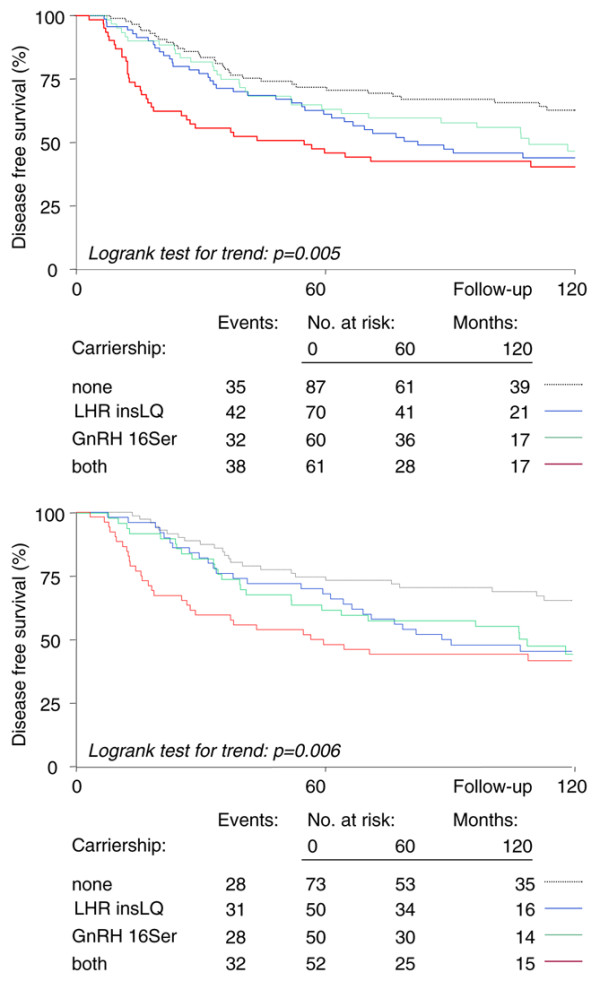
Relation of *GnRH *and *LHR *gene variants with disease-free survival. **(a) **Kaplan-Meier curves for disease free survival (DFS) comparing carriers versus non-carriers in all premenopausal breast cancer patients. DFS curves are depicted for carriers of *LHR insLQ *(blue), *GnRH 16Ser *(green) and both variants (red) versus non-carriers of both variants (gray dotted line). **(b) **Kaplan-Meier curves for DFS comparing carriers versus non-carriers in hormone receptor positive premenopausal breast cancer patients. DFS curves are depicted for carriers of *LHR insLQ *(blue), *GnRH 16Ser *(green) and both variants (red) versus non-carriers of both variants (gray dotted line).

The combination of both variants in premenopausal breast cancer patients, present in 22% of this group, resulted in a HR of 2.17 versus non-carriers of both variants (95% CI 1.36 to 3.48, *P *= 0.001; log-rank test for trend *P *= 0.001; Figure 1a and Table 2). This HR is higher than that for HER2 amplified tumors (HR of 1.61, 95% CI 1.09 to 2.39, *P *= 0.016); HER2 is amplified in about 20% of the tumors. In multivariate analysis, including the prognostic factors age, nodal status, differentiation grade, tumor size, ER status, and adjuvant therapy, the association was independent: Δχ^2 ^= 10.0 (df = 3), *P *= 0.018; HR = 2.14, 95% CI 1.32 to 3.45, *P *= 0.002. No significant interaction between the presence of the *LHR insLQ *and *GnRH 16Ser *variants was observed in these survival analyses.

We next studied the effect in hormone receptor positive patients. The combination of both variants, present in 52 out of 225 patients of this group, resulted in a HR of 2.43 versus non-carriers of both variants (95% CI 1.42 to 4.18, *P *= 0.0013; log-rank test for trend *P *= 0.0055; Figure 1b). In multivariate analysis, including the prognostic factors listed above, the association was independent: Δχ^2 ^= 8.72 (df = 3), *P *= 0.03; HR = 2.06, 95% CI 1.21 to 3.49, *P *= 0.007. No significant interaction between the presence of the *LHR insLQ *and *GnRH 16Ser *variants was observed in these survival analyses.

## Discussion

Breast cancer is a heterogeneous and complex disease. Many gene variants, acting in concert with each other and with environmental factors, may influence its susceptibility, prognosis and response to treatment [19]. In light of their possible role in the variability of estrogen exposure, variants of genes involved in the HPG axis are likely candidates to contribute to differences in clinical phenotype and outcome. In the present study we tested this hypothesis. In a genetic association approach we used stratification for ovarian activity and explored cooperative action of two HPG gene variants. We show an association between the *LHR insLQ *allele and shorter DFS in premenopausal patients, especially in the hormone receptor positive subset. A common *GnRH *gene variant, *GnRH 16Ser*, showed an association with lymph node involvement. In addition, coincident carriership of the *GnRH 16Ser *and *LHR insLQ *variants, present in almost one-quarter of the patients, resulted in a more than doubled risk of recurrence of disease in (hormone receptor positive) premenopausal patients with a long (>10 years follow-up of still living patients). Multivariate analyses showed that these associations with poor DFS were independent of known prognostic factors.

Whether variants of the *GnRH *gene differ in function remains to be elucidated. Substitution of Trp16 by the less hydrophobic serine may change the efficiency of the GnRH signal peptide. Using the same *in vitro *assay for the insLQ signal peptide variant, as described by us previously [11], we were unable to detect a difference in signal peptide efficiency between the GnRH 16Ser and GnRH 16Trp signal peptide constructs (data not shown). Furthermore, *in silico *analysis of the variants using the program SignalP 3.0 [20] did not result in any difference in signal peptide characteristics. On the other hand, Iwasaki and colleagues [13] have described an association between the *GnRH 16Trp *allele and higher bone mineral density, considered to be an indirect marker for estrogen activity, in 384 Japanese postmenopausal women; this suggests there is higher estrogen activity in women bearing this variant. In contrast, we observed a significant increased lymph node involvement and shorter DFS in Caucasian breast cancer patients carrying the other variant, the *GnRH 16Ser *allele, which we hypothesize to result from a higher level of cumulative estrogen exposure. Possible reasons for the apparent conflicting results are numerous, including differences in sample size, technical approach, and ethnicity, or so far unknown differences in the interaction of genetic and environmental factors between Japanese and Caucasian subjects. Consequently, further studies are needed to identify the exact mechanisms of the effect of the *GnRH 16Ser *polymorphism, including linkage to other polymorphisms in the *GnRH *gene that may affect regulation of expression.

There are several hypotheses as to how *GnRH *and *LHR *gene variants may affect tumor features and clinical outcome in breast cancer as demonstrated in this study. In view of the abundant data on the direct effects of GnRH modulation on sex steroid hormone-dependent cancers, a direct effect of locally produced GnRH via GnRH receptors expressed in breast cancer tissue cannot be ruled out [21-24]. Local co-expression of mRNAs for GnRH and the GnRH receptor in breast cancer tissue has been shown [25,26], and direct growth inhibition of cultured breast cancer cells have been reported as well [27]. However, to our knowledge, local production of GnRH has not been shown. Hypothalamic GnRH is unlikely to reach the breast via the peripheral circulation given its low concentration and short half-life [28]. Furthermore, the effects of GnRH agonist treatment regimens are most likely explained by down-regulation of pituitary GnRH receptors and subsequent shutdown of the HPG axis [29]. A few studies have shown LHR expression in normal and breast tumor cells. However, it is less likely that direct effects of LH explain the adverse association of *LHR insLQ *with DFS, since this was not seen in the postmenopausal patients (data not shown) in whom circulating LH levels are high.

In premenopausal women, epithelial proliferation in the non-pregnant, non-lactating breast is maximal approximately one week after ovulation in the luteal phase of the menstrual cycle. During the luteal phase, which can be considered a risky period for carcinogenesis in the breast, the corpus luteum is the main site of estrogen and progesterone hormone production, which is, in large part, dependent on LHR action. Therefore, increased cyclic hormonal stimulation of early breast cancer in women carrying activating HPG gene variants may enhance dedifferentiation and worsen prognosis. In premenopausal breast cancer patients with advanced disease, reduction of estrogen levels by a GnRH agonist in combination with the ER antagonist tamoxifen improves clinical outcome compared with the use of GnRH agonists or tamoxifen alone [29,30]. Randomized trials assessing whether the combination of a GnRH agonist with an aromatase inhibitor as adjuvant therapy improves outcome compared to treatment consisting of a GnRH agonist and tamoxifen are ongoing [31]. Regimens for endocrine therapy are still largely empirically based [32]. Recently, it has been suggested that, as for adjuvant systemic therapy, the role of genetic factors in breast cancer treatment outcome should be considered [33]. Our current study identified almost 25% of premenopausal patients with a genetic background associated with clearly significant poor outcome and hypothetically associated with altered treatment outcomes. Exploratory studies in our subset of patients that received adjuvant endocrine therapy (*n *= 4) were too underpowered to detect differences in response. Finally, it can be hypothesized that the GnRH and LHR variants may play a role in chemotherapy-induced amennorhoea [33], since 110 of the patients studied were treated with chemotherapy. Although adjuvant chemotherapy was equally divided over patients with and without polymorphisms, possible differences in the chance of ovarian failure resulting from chemotherapy may bias the outcome. However, since chemotherapy is likely to inhibit ovarian activity, this is not anticipated.

## Conclusion

We have shown a strong and independent association with DFS (HR = 2.1) in almost one-quarter of (hormone receptor positive) premenopausal women carrying *LHR insLQ *and *GnRH 16Ser *genotypes. The observations strongly suggest that the adverse outcome in patients with these variants occurs through enhanced HPG-mediated ovarian estrogen production. Prospective studies, including serum estrogen analyses, are needed. When validated in independent studies, the observed results raise the possibility that *LHR insLQ *and *GnRH 16Ser *genotyping may provide additional prognostic information for premenopausal breast cancer patients in clinical practice and may result in tailored endocrine treatments for these patients.

## Abbreviations

CI = confidence interval; df = degrees of freedom; DFS = disease free survival; ER = estrogen receptor; GnRH = gonadotropin-releasing hormone; HPG = hypothalamic-pituitary-gonadal; HR = hazard ratio; HWE = Hardy-Weinberg equilibrium; insLQ = insertion Leu-Gln; LH = luteinizing hormone; LHR = luteinizing hormone receptor; SNP = single nucleotide polymorphism.

## Competing interests

The authors declare that they have no competing interests.

## Authors' contributions

DP was involved in the acquisition of the data and drafted the manuscript. APNT and EMJJB conceived and supervised the experimental work and helped draft the manuscript. MPL carried out all statistical analysis. AGU was involved in the acquisition of the data and the drafting of the manuscript. JGMK and JAF initiated the breast tumor specimen collection program and participated in discussions on project design. HAPP reviewed the manuscript and added important intellectual content to it. All authors participated in subsequent revisions of the manuscript and approved its final version.
